# Relative importance of speech and voice features in the classification of schizophrenia and depression

**DOI:** 10.1038/s41398-023-02594-0

**Published:** 2023-09-19

**Authors:** Mark Berardi, Katharina Brosch, Julia-Katharina Pfarr, Katharina Schneider, Angela Sültmann, Florian Thomas-Odenthal, Adrian Wroblewski, Paula Usemann, Alexandra Philipsen, Udo Dannlowski, Igor Nenadić, Tilo Kircher, Axel Krug, Frederike Stein, Maria Dietrich

**Affiliations:** 1 Department of Psychiatry and Psychotherapy, University Hospital Bonn, Bonn, Germany; 2 Department of Psychiatry and Psychotherapy, University of Marburg, Marburg, Germany; 3 Center for Mind, Brain and Behavior, University of Marburg, Marburg, Germany; 4 Institute for Linguistics: General Linguistics, University of Mainz, Mainz, Germany; 5 Institute for Translational Psychiatry, University of Münster, Münster, Germany

**Keywords:** Predictive markers, Psychology

## Abstract

Speech is a promising biomarker for schizophrenia spectrum disorder (SSD) and major depressive disorder (MDD). This proof of principle study investigates previously studied speech acoustics in combination with a novel application of voice pathology features as objective and reproducible classifiers for depression, schizophrenia, and healthy controls (HC). Speech and voice features for classification were calculated from recordings of picture descriptions from 240 speech samples (20 participants with SSD, 20 with MDD, and 20 HC each with 4 samples). Binary classification support vector machine (SVM) models classified the disorder groups and HC. For each feature, the permutation feature importance was calculated, and the top 25% most important features were used to compare differences between the disorder groups and HC including correlations between the important features and symptom severity scores. Multiple kernels for SVM were tested and the pairwise models with the best performing kernel (3-degree polynomial) were highly accurate for each classification: 0.947 for HC vs. SSD, 0.920 for HC vs. MDD, and 0.932 for SSD vs. MDD. The relatively most important features were measures of articulation coordination, number of pauses per minute, and speech variability. There were moderate correlations between important features and positive symptoms for SSD. The important features suggest that speech characteristics relating to psychomotor slowing, alogia, and flat affect differ between HC, SSD, and MDD.

## Introduction

During the last century, studies have tried to disentangle major depressive disorder (MDD) and schizophrenia spectrum disorder (SSD) using transdiagnostic and multivariate approaches. However, these studies have failed to identify reproducible biomarkers for these psychiatric disorders [1–3]. Recent advances have highlighted the importance of speech features in psychiatric disorders as objective, reproducible, and time-efficient biomarkers [4–6]. In both SSD and MDD, the analysis of nonverbal speech acoustic features (i.e., prosody) is considered an encouraging prospect for developing such biomarkers [4, 5, 7].

Speech communication is the result of the coordination of over a hundred different muscles and neurobiological processes [8]. Acoustic measurement of speech can be used to observe the impacts of abnormalities on these neurobiological processes. Previous work has reported atypical acoustic measurements in both MDD and SSD. These measurements include prosody (e.g., intonation and stress), voice quality, spectral features (e.g., Mel-frequency cepstrum coefficients [MFCC]), and temporal aspects (e.g., rate, duration, number of pauses) [9]. In a meta-analysis of acoustic features in SSD, Parola and colleagues [10] reported significant group effects for speech features including decreased proportion of spoken time, decreased speech rate, and increased duration of pauses in individuals with schizophrenia. Additionally, the authors reported correlated acoustic features with clinical ratings (i.e., general psychopathology, alogia, positive and negative symptoms). Comparable to patients with SSD, decreased speech rate and increased duration of pauses have been reported in MDD as well [9, 11]. While similarities between speech and voice symptoms, particularly those related to negative symptoms, have been reported [9, 12], there are also potential distinctions between the disorders due to differences relating to positive symptoms, such as positive formal thought disorder [13, 14].

Most previous work has investigated differences in speech and voice patterns across a range of psychiatric disorders primarily through null-hypothesis significance testing (NHST [9]). While NHST has been effective in developing hypotheses related to significant and non-significant speech and voice features in psychiatric disorders, it is ultimately limited in its ability to scale with data size and complexity [15]. Recent reviews and meta-analyses of speech changes in SSD [10], MDD [11, 16], and psychiatric disorders generally [9] have suggested machine learning (ML) approaches for studying the complexity of speech in psychiatric disorders. Previous work has reported the binary classification between HC and SSD [17] or MDD [18, 19] with accuracy ranges between 72% and 91.8% [4, 10, 17]. However, few have examined both diagnoses together [12, 20].

Most ML applications in speech and voice of psychiatric disorders have focused on depression while schizophrenia is understudied [9, 10]. These applications typically have used hundreds of extracted acoustic features [12, 21]. These large feature spaces have been difficult to interpret; more so when common approaches of feature reduction (e.g., principal component analysis) have been used. These brute force black box approaches to ML have provided useful predictions for potential patients in a disorder group, but they have not provided insights into how or why the speech and voice features are contributing to the predictions [11].

Recent work has suggested study designs using interpretable machine learning (IML) that combine the applicability of null-hypothesis testing with the computational complexity of machine learning [22, 23]. IML has been either intrinsically interpretable, such as the weights of the features in a linear model, or post hoc interpretable, which requires additional models to evaluate potential structures within the explained model. One model-agnostic approach to post hoc interpretability has been permutation feature importance [24]. To optimize the interpretability, the approach should use meaningful features. Therefore, instead of a large set of abstract acoustic features, a smaller set of hypothesis-driven features is used. More targeted and theory-/hypothesis-driven research on speech and voice patterns in both MDD and SSD is timely and warranted [9, 10].

The purpose of the present proof of principle study was to investigate speech acoustics as objective and reproducible classifiers for depression and schizophrenia. The first aim of this study was to determine which speech and voice features are relatively important in the classification of SSD and MDD compared to HC. The second aim of this study was to measure how the relatively important features for disorder classification relate to symptoms of MDD and SSD. We hypothesized that (1) speech samples from patients with MDD and SSD will be accurately classified from healthy controls and (2) the important features will correlate with symptom severity scores related to depression and negative and positive symptoms in SSD. The relative importance of the interpretable features and the correlations with the symptom scores will provide a basis for inference on the differences in speech patterns between patients with SSD and MDD.

## Methods

### Participants

Participants were selected from a supplemental study of the longitudinal Marburg/Münster Affective Disorders Cohort Study [25]. This longitudinal study started in 2014 aiming at the neurobiological analysis of the pathophysiology and course of affective disorders (see Kircher et al. for more details) [25]. For the present cross-sectional study, participants were included regardless of time point in the larger study (i.e., baseline and follow-up after two and five years) and consisted of 20 patients suffering from DSM-IV SSD, 20 from MDD, as well as 20 HC participants. While participants were from different time points, each participant was only assessed once. As the pool of available patients with SSD was the smallest, a random group of 20 participants was first chosen from this group. Then MDD patients and HC were 1:1 matched by age and sex of the SSD group using MatchIt in R [26].

Exclusion criteria were current or past alcohol or drug dependency, traumatic brain injury, neurological diseases, and a verbal IQ below 80 (see Kircher et al. for more details) [25]. Further, HC were excluded if they had a first relative that had been diagnosed with any psychiatric disorder. During a semi-structured interview, clinical diagnoses were assessed according to the German version of the Structured Clinical Interview for DSM-IV (SKID-I) [27] and additional rater-based psychopathological scales. Interrater reliability was assessed with the interclass coefficient, achieving good reliability of *r* > 0.86 in all clinical assessments.

The ethics committee of the University of Marburg approved the study (AZ07–2014) according to the Declaration of Helsinki, and participants gave written informed consent.

Descriptive characteristics of the three groups are shown in Table 1. One participant in the SSD group was replaced because a majority of the speech sample (>75% of the recording duration) contained more noise than speech. This was a result of a combination of high background noise (both stationary and transient) and low speech volume from the participant.

**Table 1:** Descriptive characteristics of participants (HC, healthy controls; MDD, major depressive disorder; SSD, schizophrenia spectrum disorder).

	HC (*n* = 20)	MDD (*n* = 20)	SSD (*n* = 20)	*P*
Age (years)	39.3 (12.7, range 24–60)	41.5 (13.2, range 21–64)	41.6 (11.6, range 20–60)	0.799
Sex	M = 13	M = 10	M = 14	0.400
	F = 7	F = 10	F = 6	
Years of education	15.4 (2.4)	12.3 (2.3)	12.1 (2.3)	<0.001
Age of onset	—	26 (13.7)	18.69 (7.5)	0.067
Duration of illness (years)	—	16.3 (12.4)	18.7 (7.5)	0.28
Duration of hospitalizations (weeks)	—	11.3 (18.2)	22.7 (29.6)	0.166
Antidepressant intake *n*, (%)	—	11 (55)	4 (20)	0.048
Antipsychotic intake *n*, (%)	—	1 (5)	12 (60)	<0.001
Mood stabilizer intake *n*, (%)		0 (0)	1 (5)	1
HAM-A	1.20 (1.68)	8.40 (7.70)	9.90 (10.10)	<0.001
HAM-D	0.82 (1.78)	6.15 (7.26)	8.35 (8.42)	0.005
SANS	0.12 (0.49)	6.2 (8.5)	17.3 (11.39)	<0.001
SANS alogia subscale	0 (0)	1.06 (12.26)	2.35 (2.50)	<0.001
SAPS	0 (0)	1.47 (2.15)	19.32 (15.43)	<0.001
SAPS FTD subscale	0 (0)	1.67 (2.50)	9.35 (9.20)	<0.001
GAF	92.4 (7.0)	65.7 (12.8)	47.9 (17.8)	<0.001

*M* male, *F* female, HAM-A Hamilton Anxiety Rating Scale, HAM-D Hamilton Depression Scale, SANS Scale for the Assessment of Negative Symptoms, SAPS Scale for the Assessment of Positive Symptoms, FTD formal thought disorder, GAF Global Assessment of Functioning.

### Picture description task

To elicit spontaneous speech, a picture description task based on the Thematic Apperception Test (TAT) was used [28]. From the TAT, pictures 1, 2, 4, and 6 were displayed in front of the participants individually and in the same order. Participants were asked to describe each picture, express thoughts, or tell a story for a total of three minutes per picture. The speech was recorded using a digital voice recorder (Olympus WS-853, OM Digital Solutions GmbH, Hamburg, Germany) placed in the middle of the table facing the participant. The average distance between the recorder and mouth of the participants was 35 cm.

### Segmentation

Each picture description was segmented, resulting in four speech samples per participant. For each speech sample, the examiner’s speech and instances of excessive background noise were manually removed by the first author (M.B.). See Supplemental Material Table S1 for further preprocessing details specific to the calculation of each feature.

### Feature extraction

Prior to classification, all features were calculated from the individual speech samples. The set of features was chosen from those reported in Low, Bentley, and Ghosh [9] that could be used as sample-level features. These features included speech tempo features (speech rate, articulation rate, talking rate), speech pause features (pause duration, pause duration standard deviation [SD], and pause rate), prosodic intonation features (fundamental frequency [*f*_o_] SD, kurtosis, and skewness), prosodic stress features (intensity SD, kurtosis, skewness, and energy velocity), and speech spectrum features (mean MFFC for coefficients 1 through 13). Additionally, other potentially useful features from other publications were added. These included pauses per minute (PPM) [7] and articulation coordination features (three vocal-tract-variable-based articulation coordination features [ACF1, ACF2, ACF3]) [29]. Finally, while not previously reported a novel addition of vocal quality features (mean smoothed cepstral peak prominence [CPPs], CPPs SD, kurtosis, and skewness, low-to-high ratio mean [LHR], LHR SD, kurtosis, and skewness) were added to test the impact of clinical voice pathology measures. See supplemental Table S1 for detailed explanations, methods on feature calculations, and references for rationale of inclusion.

### Model selection

Three pairwise classification models were used to compare differences in feature importance. Following the work by Espinola, Gomes, Pereira, and dos Santos [17] who used similar features for the classification of schizophrenia and found support vector machines (SVM) provided the best performance, here the classification models were SVM with three polynomial kernels (*n* = 1, 2, 3). Additionally, five-fold cross-validation was used for validation accuracy. To accomplish this, for each model standardized data were randomly divided into five equal-sized folds while maintaining class balance and participant assignment for each fold. Then the model was trained in five iterations, the first iteration used the first fold as the validation set and the remaining four folds were combined and used as the training set. The model was trained and validated, then iterated four more times using each fold as a validation set. After completing the five iterations, the performance metrics (accuracy, precision, recall, and F1-score) were averaged to provide an overall assessment of the model’s performance. The models’ box constraints were set using Bayesian hyperparameter optimization with an expected improvement acquisition function and the kernels were allowed to auto-scale. The machine learning pipeline was implemented in MATLAB (2021b, MathWorks, Natwick, Massachusetts) using the Statistics and Machine Learning Toolbox and the code is available upon request from the corresponding author.

### Feature importance

Feature importance through permutation was computed for each model. For each feature, the respective trained model was tested on a randomized permutation of the values. The difference between the testing accuracies with and without permutation was the feature importance score (FI). This was repeated 20 times and an average accuracy was computed. To mitigate bias from the random nature of the cross-validation in the machine learning and permutation feature importance testing, the entire pipeline including the randomized train-test split was repeated 100 times and average performance metrics and feature importance scores were computed.

### Descriptive statistics

The statistical relationships between the most important features (top 25%) and the three classification groups were calculated. For important features, the percent difference between the HC group and the two clinical groups was calculated and tested for statistical significance with an ANOVA at an alpha level of α = 0.05 with Bonferroni corrections. Prior to ANOVA testing, the data was checked for the assumptions of normality and equal variances (Levene’s test). If normality could not be assumed, non-parametric Mann Whitney U tests were used. When normally distributed but equal variances could not be assumed, Welch-ANOVA tests were used. Additionally, two-tailed Pearson correlations were calculated between the important features and symptom severity scores from the Hamilton Depression Rating Scale (HAM-D) [30], Scale for the Assessment of Negative Symptoms (SANS) [31], Scale for the Assessment of Positive Symptoms (SAPS), and the subscales for alogia, flat affect, and formal thought disorder (FTD) [32]. The two-sided Pearson correlations were tested for significance at an alpha level of α = 0.05 with Bonferroni corrections.

## Results

### Model accuracies

The performance metrics (validation accuracy, precision, recall, and F1 score) from the repetitions were averaged for each model. Table 2 summarizes these metrics for each pairwise comparison for the three degrees of polynomial kernel. The box constraints for each model are in Table S2.

**Table 2:** Summary of 5-fold cross-validation accuracy, precision, recall, and F1 score for each classification model.

Model	Pairwise comparison	Accuracy	Precision	Recall	F1 Score
SVM Linear	HC and SSD	0.793	0.798	0.791	0.794
	HC and MDD	0.736	0.718	0.742	0.730
	SSD and MDD	0.653	0.659	0.656	0.657
SVM 2-degree Polynomial	HC and SSD	0.933	0.944	0.925	0.934
	HC and MDD	0.900	0.895	0.905	0.900
	SSD and MDD	0.916	0.923	0.912	0.918
SVM 3-degree Polynomial	HC and SSD	0.947	0.965	0.933	0.949
	HC and MDD	0.920	0.921	0.920	0.920
	SSD and MDD	0.932	0.943	0.924	0.933

*SVM* support vector machine, *HC* healthy control, *SSD* schizophrenia spectrum disorder, *MDD* major depressive disorder.

### Feature importance

**Fig. 1:**
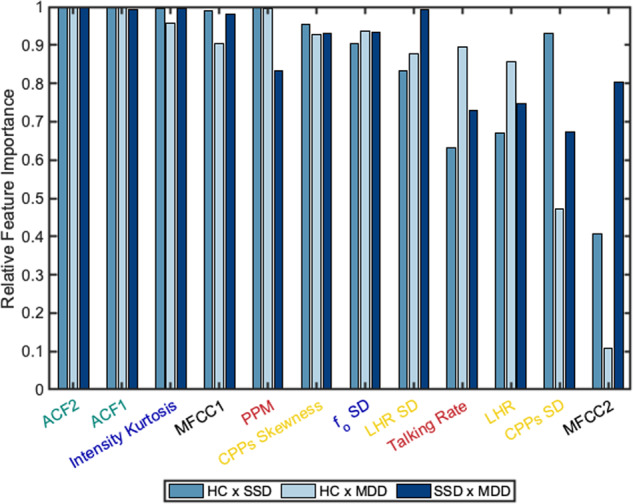
Comparison of the top 25% of features ranked by aggregate feature importance across all pairwise models with the 3-degree SVM polynomial kernel. Feature importance was computed as the post hoc permutation feature importance. For each model, there were twenty participants per group (HC healthy controls, SSD schizophrenia spectrum disorder, MDD major depressive disorder) and four speech samples per participant.

For each ML classification model with each kernel, the relative importance of the features was computed. Figure 1 shows the features that were in the top 25% of important features for any of the three pairwise models with the best performing model, the SVM with 3-degree polynomial kernel. See supplementary material figures S1-S5 for relative importance of all features for each pairwise model with each kernel as well as the top 25% of important features for the models with the linear and 2-degree polynomial SVM kernels. The same features were included in the top 25% for each SVM kernel. Of the top 25% of features, the features that were relatively important (top 25%) for all pairwise models included (in order of total relative importance for the best performing kernel) ACF2, ACF1, intensity kurtosis, MFCC1, PPM, CPPs skewness, *f*_o_ SD, LHR SD, and LHR. Three features that were uniquely important (top 25% for only one model) were CPPs SD for HC x SSD, talking rate for HC x MDD, and MFCC2 for SSD x MDD.

### Descriptive statistics

For each of the most important features, the differences between HC and SSD, as well as HC and MDD were calculated. Table 3 summarizes the percent difference for each measure, the *F-*statistic and the *P*-value for the corresponding ANOVA test. The data met the assumption of normality. Additionally, for the group comparison of MDD and HC, all features met the assumption of equal variance except LHR SD. For the group comparison of SSD and HC, all features met the assumption of equal variance except ACF1, ACF2, and *f*_o_ SD.

**Table 3:** Provides a summary of the mean difference (percent change), the *F-*statistic, and the *P*-value for the corresponding ANOVA test for the differences of the top 25% of important features between the three classification groups, healthy controls (HC), major depressive disorder (MDD), and schizophrenia spectrum disorder (SSD).

Feature	Group comparison	Mean difference (%)	*F*	*P*
ACF2	MDD-HC	−4.7	7.29	**0.008**
	SSD-HC	−4.1	4.14	**0.044**
ACF1	MDD-HC	3.8	6.73	**0.010**
	SSD-HC	3.6	4.65	**0.033**
Intensity kurtosis	MDD-HC	29.7	7.83	**0.006**
	SSD-HC	13.3	1.55	0.215
MFCC1	MDD-HC	−2.6	7.16	**0.008**
	SSD-HC	−3.1	11.10	**0.001**
PPM	MDD-HC	−16.3	19.47	**<0.001**
	SSD-HC	−17.2	20.81	**<0.001**
CPPs skewness	MDD-HC	345.4	5.39	**0.022**
	SSD-HC	28.2	0.04	0.845
*f*_o_ SD	MDD-HC	−1.4	0.04	0.849
	SSD-HC	−14.5	6.56	**0.011**
LHR SD	MDD-HC	−2.3	1.35	0.248
	SSD-HC	−6.7	7.37	**0.007**
Talking rate	MDD-HC	−8.0	5.83	**0.017**
	SSD-HC	−10.2	8.97	**0.003**
LHR	MDD-HC	−5.8	6.76	**0.010**
	SSD-HC	−2.8	1.29	0.257
CPPs SD	MDD-HC	−10.3	22.95	**<0.001**
	SSD-HC	−7.7	11.29	**0.001**
MFCC2	MDD-HC	−1.7	0.45	0.503
	SSD-HC	3.6	1.58	0.211

*P*-values that are significant at α = 0.05 are bolded.

The acoustic features that had statistically significant (*P* < 0.05) changes from HC to both patient groups were ACF2, ACF1, MFCC1, PPM, talking rate, and CPPs SD. The acoustic features that only had statistically significant changes from HC to MDD were intensity kurtosis, CPPs skewness, and LHR. The acoustic features that only had statistically significant changes from HC to SSD were *f*_o_ SD and LHR SD. MFCC2 had no statistical differences between HC and either patient group.

In addition to differences of the acoustic features between the patient groups, the correlations between the acoustic features and symptom severity scores were computed. Table 4 summarizes two-tailed Pearson correlations between the top 25% important features and the symptom severity scores from the HAM-D as well as SANS and SAPS with their subscales for alogia, flat affect, and FTD. Following multiple testing corrections, moderate positive correlations were found for LHR SD and HAMD (*r* = 0.50) and SAPS (*r* = 0.40) for SSD. There were also moderate positive correlations between CPPs Skewness and SANS (*r* = 0.47) and SAPS FTS (*r* = 0.42) for SSD. There was a negative correlation between intensity kurtosis and SAPS for SSD (*r* = -0.39). Finally, there was a moderate positive correlation for MFCC1 (*r* = 0.46) and a moderate negative correlation for PPM (*r* = -0.42) with SAPS FTD for SSD.

**Table 4:** Two-tailed Pearson correlations between the top 25% important features and symptom severity scores for the two patient groups, major depressive disorder (MDD) and schizophrenia spectrum disorder (SSD).

	HAMD		SANS		SAPS		SANS Alogia		SANS flat affect		SAPS FTD	
	SSD	MDD	SSD	MDD	SSD	MDD	SSD	MDD	SSD	MDD	SSD	MDD
ACF2	−0.02	−0.12	−0.21	0.13	0.03	0.15	−0.19	0.39	−0.12	0.20	−0.26	0.04
ACF1	−0.03	−0.06	0.13	−0.15	0.06	−0.18	0.24	−0.12	−0.05	−0.32	0.19	−0.09
Intensity Kurtosis	−0.32	−0.06	−0.12	0.12	**−0.39**	0.14	−0.33	−0.14	−0.14	−0.29	−0.24	0.06
MFCC1	0.01	0.04	−0.09	−0.11	0.27	−0.15	0.16	0.25	−0.05	−0.01	**0.46**	0.01
PPM	−0.13	0.31	−0.35	−0.10	−0.06	0.03	−0.24	−0.23	−0.26	−0.19	**−0.42**	0.16
CPPs Skewness	0.08	−0.32	**0.47**	0.01	0.05	−0.10	−0.03	0.25	0.37	−0.08	**0.42**	−0.22
*f*_o_ SD	0.01	0.08	0.08	−0.20	−0.08	−0.14	−0.12	−0.28	0.01	0.08	−0.03	0.02
LHR SD	**0.50**	−0.31	0.03	−0.18	**0.40**	−0.17	0.16	−0.12	−0.05	0.29	−0.25	−0.20
Talking Rate	−0.11	0.10	−0.05	0.17	−0.06	0.15	−0.30	−0.04	0.18	−0.06	0.23	−0.02
LHR	0.06	0.00	0.03	−0.32	0.09	−0.26	0.24	−0.08	0.22	0.18	−0.02	0.05
CPPs SD	0.01	−0.12	−0.09	0.08	0.07	0.14	−0.08	−0.02	−0.29	0.19	−0.36	−0.08
MFCC2	−0.02	0.11	−0.05	−0.03	−0.04	0.04	0.09	0.01	0.08	0.26	−0.18	0.16

*HAM-D* Hamilton Depression Scale, *SANS* Scale for the Assessment of Negative Symptoms, *SAPS* Scale for the Assessment of Positive Symptoms, *FTD* formal thought disorder.

Correlations that are significant at α = 0.05 with Bonferroni correction are bolded.

## Discussion

The important differences in speech patterns between the patient groups provide evidence for the use of speech as a potential biomarker for psychiatric disorders, specifically SSD and MDD. In this proof of principle study, pairwise SVM learning models, which used a limited number of hypothesis-driven speech and voice features, classified speech samples between HC and two different patient groups—SSD and MDD. All three models had high testing accuracies ( >0.90) for the 2-degree and 3-degree polynomial kernels. This supports the hypothesis that the models could accurately classify speech samples among SSD, MDD, and HC groups with a set of interpretable features. The secondary hypothesis that important features would correlate with symptom scores was partially supported by moderate correlations for SSD speech samples.

### Classification performance

The testing accuracies of the classification of SSD were slightly higher than previously reported. A systematic review [10] reported five ML studies classifying schizophrenia with acoustic features and reported accuracies from 0.75 to 0.875. Siriwardena, Espy-Wilson, Kitchen, and Kelly [33] reported classification accuracy using the ACFs of 0.722 but showed increased accuracy of 0.833 when using a multimodal approach that included features derived from facial images. Espinola, Gomes, Pereira, and dos Santos [17] had a similar sample size (*n* = 20 schizophrenia) and feature set (33 extracted acoustic features) and reported similar performance changes with different polynomial kernels for SVM classification of HC vs. schizophrenia (0.78, 0.89, and 0.90 for linear, 2-degree polynomial, and 3-degree polynomial respectively).

One potential reason for the slightly higher performance in the present study is the inclusion of a variety of features at the sample level that represent varied paralinguistic aspects of speech. Another potential reason is the use of the picture-description task. The aforementioned studies contained either reading samples or recorded interviews. The speech samples here allowed for structured extemporaneous speech production, which preserves altered speech and language production but also is consistent enough to be comparable across participants.

### Important features across all models

Post hoc model-agnostic evaluation of the importance of the features shows that some of the features were important for all models. Two of these features were ACF1 and ACF2. These two features represent the complexity of articulation of the speech. Additionally, both features were significantly (*p* < 0.05) different between each disorder group and HC (see Table 3). There was an increase in ACF1 and a decrease in ACF2 for both disorder groups compared to the controls. This relationship is consistent with previously reported work using ACFs and classifying the speech of people with depression [29, 34]. However, an inverse relationship was expected for speech in schizophrenia [35], specifically with subjects with strong positive symptoms [33]. Table 4 shows that ACF2 has an inverse relationship between patients with MDD on the SANS alogia subscale and patients with SSD on the SAPS FTD subscale. These relationships are consistent with previous work and suggest competing influences on the articulation coordination in SSD implying a potential value in ACF as a speech feature to distinguish positive and negative symptoms.

Differences in articulatory coordination between patient groups related to negative symptoms are consistent with psychomotor slowing, which would directly affect speech kinematics [36]. Psychomotor slowing has been described as psychomotor retardation in MDD [37] and psychomotor poverty in SSD [38]. The differential contribution of cognitive and motor deficits to the slowing is of theoretical interest as it should provide further insights into the pathophysiology of SSD. Previous work has suggested that psychomotor slowing may serve as an endophenotype and biomarker of SSD with planning and response selection particularly affected [39]. As neurobiological regions for motor planning intersect with regions for (emotional) voice and speech production, the effects on speech production are not surprising [40].

Another feature that was important for all three models was PPM. Additionally, PPM has similar percent decreases ( >16%) from HC in both patient groups (see Table 3) and significant moderate correlation (*r* = -0.42; *P* < 0.001) with the SAPS FTD subscale (see Table 4), which is consistent with previous work [7, 41].

Other major features that were important for all models relate to measures of voice and speech variability (intensity kurtosis, CPPs skewness, *f*_o_ SD). CPPs skewness and *f*_o_ SD had correlations with SANS alogia in MDD and intensity kurtosis had a moderate correlation with SANS alogia in SSD. This result is not surprising as the negative symptoms of MDD and SSD can manifest as a lack of variability or dynamics of speech [42–44]. Table 3 shows that *f*_o_ SD decreased with the patient groups and the intensity kurtosis increased, which can be interpreted as an increase in the number of data points away from the mean (e.g. outliers). In other words, this speech pattern is more consistent with monotone speech than typical speech, with occasional brief changes in loudness.

### Transdiagnostic similarities and differences

The two patient groups shared similar differences from HC in the important features. Both groups had significant changes (*p* < 0.05) and in the same direction for the ACF features, MFCC1, PPM, and talking rate (see Table 3). However, there was a unique difference between MDD and SSD patients in speech variability, specifically for *f*_o_ SD and LHR SD. For both features, there was reduced variability compared to controls but for SSD the difference was greater and significant compared to controls (see Table 3). As previously mentioned, the lack of variability and dynamics of speech is expected. In the present data set it is more pronounced for SSD.

### Voice pathology features

Our study uniquely sheds a light on voice pathology features largely neglected in previous research on vocal features in psychiatric disorders. Voice pathology features such as the dysphonia measures CPPs and LHR [45], which have not been previously used in classification, were relatively important in the differentiation between MDD and SSD. These features highlight degradations in voice quality, which may stem from physiological aspects of vocal fold vibration including diminished mucosal hydration (vocal tract drying) to altered posturing of the vocal folds leading to hypoadduction [46]. Lowered voice fundamental frequency and intensity have been typical in MDD [46]. Moreover, introversion and neuroticism have been found to be elevated in MDD compared to controls [47], which is a personality mix that is thought to contribute to inhibited voice production in novel or threatening situations [48]. Including voice pathology features may help to better classify between MDD and SSD groups as LHR SD was the most important feature for the SSD vs. MDD model and LHR was uniquely important for the MDD binary models. Moreover, cepstral-spectral measures have held a significant advantage over so-called traditional perturbation measures of voice such as jitter and shimmer [49]. They can be applied to running speech as well [45, 50]. Typically, classification studies focus on one particular task, but in the interest of uncovering the pathophysiology of SSD a set of tasks with differing speech complexity (sustained vowels to spontaneous speech, parsing voice and speech) and varying cognitive, emotional, and social demands will be revealing [10]. Systematically studying speech and voice features in research across psychiatric disorders also aligns with a call to carefully study motor behavior in general as a key to better understanding underlying mechanisms across psychiatric disorders [51]. Specifically, deeper insights into the mechanisms for altered speech and voice motor behavior in SSD must be pursued as proposed by Parola et al. [10]. who have suggested looking at auditory processing, pitch control, neuromotor disorders, and antipsychotic medication.

### Medication use

Medication use has had potential impacts on the voice. A little over half of the participants in the MDD group used antidepressants whereas a little over half of the participants in the SSD group used antipsychotic medications. Medication use was not specifically controlled for due to its differing distribution in our transdiagnostic sample and therefore differential effects on acoustic features and psychopathology. Additionally, given the nearly equal distribution of medication use in the patient groups, significant group effects due to medication are minimized in the classification. Supplemental Table S3 includes a report for one-way ANOVA tests on medication use (antidepressants for MDD and antipsychotics for SSD) in the patient groups for all of the speech features. Two notable results from this table include a significant decrease in CPPs for medication use in MDD, which is consistent with previous work that showed a relationship between antidepressants and voice quality measured by CPPs [52]. Additionally, there were increases in *f*_o_ SD for both patient groups with medication, which suggests an effect of medication to dampen the impact of psychiatric disorders on the monotonicity of speech.

### Limitations

The speech samples used were from patients in inpatient and outpatient clinical settings. As a result, there are inherent limitations such as the participants being in various stages of illness, which can have varying influences on speech and voice parameters. For example, positive symptoms dominate in the acute phase whereas negative symptoms dominate in the chronic phase. Additionally, while the groups were closely matched on age and sex, other confounders are possible. For example, there was a difference in education level between the groups which has been previously shown to be associated with verbal performance and processing acoustic information [53]. A single modality of speech task was used in the study (e.g., continuous speech from picture descriptions). Other speech tasks such as sustained vowels have been used in previous work and have provided other insights to vocal function [9]. Furthermore, speech tasks with varying levels of linguistic complexity could elicit additional acoustic abnormalities that could be used to improve the classification. Additionally, the study is limited through using a single language. Recent research has revealed significant variations in vocal patterns in SSD when comparing different languages [41], therefore it is important to expand the work to include a variety of speech and languages. Finally, the study is limited by the number of participants. A larger sample size and an independent testing set would improve the confidence in the machine learning results.

### Future directions

One advantage of the model-agnostic feature importance approach is that it allows for the scalability of future work in terms of data size and complexity. One potential future application is multi-class classification, which presents a difficult but potentially insightful challenge [54]. Additionally, future work looks to expand beyond binary classification of the disorders towards severity of symptoms relating to the disorders and subtypes. In these cases, feature importance related to the severity of the symptoms can be computed to investigate the potential relationships between voice and speech and these symptoms beyond correlations. This has the potential to lead towards tracking symptom severity and changes in patients. This approach takes advantage of the benefits of using speech features for analysis as they are easy to obtain, in particular in voice centers or laryngology clinics as suggested by Low, Bentley, and Ghosh [9], which could facilitate the interdisciplinary automated assessment of psychiatric disorders within ethical limits. Additionally, psychiatric care facilities can easily implement speech sampling to be used for future application such as monitoring. However, replication in a large, independent sample is essential for successful future application.

## Conclusion

Speech recordings offer a noninvasive and inexpensive evaluation of psychiatric disorders such as SSD and MDD. To determine potential differences in speech patterns between HC and patients with SSD or MDD, classification models with interpretable features were developed and applied to speech recordings of these groups. A model-agnostic approach to feature importance was used to determine which of the features were most important to the classification of the psychiatric disorders. These important features were compared across the patient groups and symptom scores and suggest differences in how symptoms manifest in speech. Aspects of the articulatory coordination and variability of speech were most important in classifying clinical diagnoses and have the potential to serve as speech biomarkers. Future work can expand these findings in more transdiagnostic studies, both with multimodal data and with more specific symptoms related to the psychiatric disorders.

### Supplementary information

Supplemental Material

Figure_S1

Figure_S2

Figure_S3

Figure_S4

Figure_S5

## Data Availability

The data and code supporting the findings of this study can be accessed by contacting the corresponding author (MB).
