# Supplementary material for: Relative importance of speech and voice features in the classification of schizophrenia and depression
Source: Transl Psychiatry. 2023 Sep 19;13:298. doi: 10.1038/s41398-023-02594-0 (PMC10509176; doi:10.1038/s41398-023-02594-0)
Supplement: Supplementary file 1 — Supplemental Material [file 41398_2023_2594_MOESM1_ESM.docx]

# Supplemental Material

Table S1. List of features used in the machine learning classification with descriptions and further details on the rationale for inclusion and detailed methodology of calculation.

| Feature | Description | Software | | References |
| --- | --- | --- | --- | --- |
| *Speech Tempo Features* |  |  |  | |
| Speech rate | Number of speech utterances per second for the speech sample with pauses^a^ | MATLAB | Low, Bentley,Ghosh (2020)^1^ | |
| Articulation rate | Number of speech utterances per second for the speech sample without pauses^a^ | MATLAB | Low, Bentley, Ghosh (2020)^1^ | |
| Talking rate | Total duration of the speech segments after pauses are removed divided by the total length of the speech segment with pauses^a^ | MATLAB | Low, Bentley, Ghosh (2020)^1^ | |
| *Speech Pauses Features* |  |  |  | |
| PPM | Number of pauses divided by the total length of the speech segment with pauses in minutes^a^ | MATLAB | Matsumoto, et al (2013)^2^ | |
| Pause duration | Average length of the pause segments in the speech sample^a^ | MATLAB | Low, Bentley, Ghosh (2020)^1^ | |
| Pause duration SD | Standard deviation of length of the pause segments in the speech sample^a^ | MATLAB | Low, Bentley, Ghosh (2020)^1^ | |
| Pause rate | Total duration of the pauses in the speech sample divided by the total length of the speech segment with pauses^a^ | MATLAB | Low, Bentley, Ghosh (2020)^1^ | |
| *Prosodic Intonation Features* |  |  |  | |
| *f*_o_ SD | Fundamental frequency standard deviation^b^ | Praat | Low, Bentley, Ghosh (2020)^1^ | |
| *f*_o_ kurtosis | Fundamental frequency kurtosis*^b^ | Praat |  | |
| *f*_o_ skewness | Fundamental frequency skewness*^b^ | Praat |  | |
| *f*_o_ velocity | Average magnitude of the first central difference of fundamental frequency over time | Praat & MATLAB | Qutieri, Malyska (2012)^3^ | |
| *Prosodic Stress Features* |  |  |  | |
| Intensity SD | Intensity standard deviation^c^ | MATLAB | Low, Bentley, Ghosh (2020)^1^ | |
| Intensity kurtosis | Intensity kurtosis*^c^ | MATLAB |  | |
| Intensity skewness | Intensity skewness*^c^ | MATLAB |  | |
| Energy velocity | Average magnitude central difference of the intensity over time | MATLAB | Low, Bentley, Ghosh (2020);^1^  Qutieri, Malyska (2012)^3^ | |
| *Voice Quality Features* |  |  |  | |
| CPPs | Average smoothed cepstral peak prominence^d^ | Praat | Heman-Ackah, et al (2003);^4^ Silva, et al (2021)^5^ | |
| CPPs SD | Smoothed cepstral peak prominence standard deviation*^d^ | Praat |  | |
| CPPs kurtosis | Smoothed cepstral peak prominence kurtosis*^d^ | Praat |  | |
| CPPs skewness | Smoothed cepstral peak prominence skewness*^d^ | Praat |  | |
| LHR | Average low-to-high ratio^e^ | MATLAB | Silva, et al (2021);^5^ Awan, Roy (2006)^6^ | |
| LHR SD | Low-to-high ratio standard deviation*^e^ | MATLAB |  | |
| LHR kurtosis | Low-to-high ratio kurtosis*^e^ | MATLAB |  | |
| LHR skewness | Low-to-high ratio skewness*^e^ | MATLAB |  | |
| *Spectral Features* |  |  |  | |
| MFCC (1-13) | Average Mel-frequency cepstral coefficients for Mel-frequency bands *N* = [1,2,3…13]^f^ | MATLAB | Low, Bentley, Ghosh (2020)^1^ | |
| *Articulation Coordination Features* |  |  |  | |
| ACF1 | Average of the first 5 eigenvalues of the articulation coordination matrix of the vocal tract variables^g^ | Python | Espy-Wilson, et al (2019)^7^ | |
| ACF2 | Average of the middle 5 eigenvalues of the articulation coordination matrix of the vocal tract variables^g^ | Python | Espy-Wilson, et al (2019)^7^ | |
| ACF3 | Average of the last 5 eigenvalues of the articulation coordination matrix of the vocal tract variables^g^ | Python | Espy-Wilson, et al (2019)^7^ | |

Notes:

* Statistical moments (standard deviation [SD], kurtosis, and skewness) were included to represent the distribution of the frame-level acoustic features on the sample-level scale

^a^ MATLAB’s (2021b, Natwick, Massachusettes: The MathWorks Inc.) voice activity detection (VAD)^8^ was used to identify and remove pauses and silences from the speech samples. The speech prosody features were computed from the speech sample durations measured during this pause removal process using MATLAB’s VAD. The speech utterance counts were from the transcription of the speech samples. Speech samples were transcribed by trained linguistic staff that was blind to the clinical diagnosis using the f4transkript software (Dr. Dresing & Pehl GmbH, Marburg, Germany). Transcription was based on the guidelines of the Gesprächsanalytischen Transkriptionssystems (GAT).^9^

^b^ Voice fundamental frequency (*f*_o_) is the acoustic measure that correlates with the perception of pitch and measures the frequency of vibration of the vocal folds during speech. Since there can be a wide range of average *f*_o_ —particularly between female and male voices—a two-step process for measuring *f*_o_ was used. For both steps, Praat (v6.2)^10^ was used to compute the *f*_o_ across the speech sample (using cross-correlation and 10 ms window size). For the first step, a pitch range of 75 Hz to 500 Hz was used. From these measurements, the 1st and 3rd quartile were calculated. For the second step, an individualized *f*_o_ range of 0.75 times the 1st quartile to 1.5 times the 3rd quartile was used. After this second calculation of *f*_o_, statistical moments of the distribution (SD, kurtosis, and skewness) were computed.

^c^ Intensity was computed as root-mean square of the amplitude of the signal at 10 ms windows with 50% overlap across the speech sample. Statistical moments (SD, kurtosis, and skewness) of the intensity distribution were computed.

^d^ CPPs was calculated through Praat using an 87 ms window with 79% overlap.^11^ Additionally, as part of computing CPPs in Praat, the non-voicing segments of the speech were removed.^12^ Statistical moments (mean, SD, kurtosis, and skewness) were calculated from this distribution.

^e^ The LHR is the ratio between acoustic energy above and below 4 kHz. Here the LHR was computed using a 20 ms window with 75% overlap.^6^ Statistical moments (mean, SD, kurtosis, and skewness) for both CPPs and LHR distributions were computed.

^f^ Thirteen MFCCs were computed using MATLAB’s Auditory Toolbox.^13^ Typically, MFCC is used as a frame-level feature when used for speech classification.^14^ Since the current experiment is using sample-level features (in particular the speech tempo and pauses features), an average of the MFCCs across the entire sample is used.

^g^ The articulation coordination features (ACF) were computed from estimated vocal tract constriction variables from a pretrained neural network.^7,15^ The network estimated six tract variables (lip aperture, lip protrusion, tongue tip construction degree, tongue tip constriction location, tongue body constriction degree, and tongue body constriction location) from MFCCs. The ACFs are computed from the eigenvector of the coordination matrix of these tract variables.

Table S2. Box constraints for each pairwise model. These were set using Bayesian hyperparameter optimization with an expected improvement acquisition function.

|  | **Box Constraints** | | |
| --- | --- | --- | --- |
| **Model** | **SVM linear** | **SVM 2-degree polynomial** | **SVM 3-degree polynomial** |
| **HC and SSD** | 2.08 | 2.70 | 2.12 |
| **HC and MDD** | 3.96 | 1.14 | 2.19 |
| **SSD and MDD** | 2.27 | 1.34 | 1.25 |
|  |  |  |  |

Note: SVM = support vector machine, HC = healthy control, SSD = schizophrenia spectrum disorder, MDD = major depressive disorder

Table S3. Summary of differences in speech features between groups with and without medication for both major depressive disorder (MDD) and schizophrenia spectrum disorder (SSD). For MDD, the with medication group are patients currently using antidepressants (n = 10). For SSD, the with medication group are patients currently using antipsychotics (n = 12). The comparisons were made using one-way ANOVA tests in SPSS with α = .05.^16^

|  | **Major Depressive Disorder** | | | **Schizophrenia Spectrum Disorder** | | |
| --- | --- | --- | --- | --- | --- | --- |
| **Speech Feature** | **Without Medication; Mean (SD)** | **With Medication; Mean (SD)** | **Difference Significance;  *F* (*P*)** | **Without Medication; Mean (SD)** | **With Medication; Mean (SD)** | **Difference Significance;  *F* (*P*)** |
| **ACF1** | 6.80 (0.50) | 6.73 (0.80) | 0.24 (.622) | 6.90 (0.69) | 6.64 (0.85) | 2.12 (.149) |
| **ACF2** | **0.28 (0.03)** | **0.29 (0.04)** | **4.24 (.043)** | 0.28 (0.03) | 0.29 (0.05) | 2.17 (.144) |
| **ACF3** | **0.02 (0.00)** | **0.02 (0.00)** | **5.26 (.024)** | **0.02 (0.00)** | **0.02 (0.00)** | **4.64 (.034)** |
| **Articulation rate** | **3.10 (0.56)** | **3.46 (0.50)** | **9.12 (.003)** | 3.37 (0.78) | 3.32 (0.90) | 0.08 (.785) |
| **CPPs kurtosis** | 2.17 (0.26) | 2.25 (0.33) | 1.65 (.202) | **2.09 (0.27)** | **2.25 (0.30)** | **5.79 (.019)** |
| **CPPs** | **6.34 (1.51)** | **5.21 (1.89)** | **8.81 (.004)** | 6.50 (1.73) | 6.29 (2.05) | 0.22 (.637) |
| **CPPs SD** | 4.95 (0.84) | 4.90 (0.66) | 0.09 (.769) | **5.32 (0.62)** | **4.89 (0.94)** | **5.34 (.023)** |
| **CPPs skewness** | **-0.03 (0.19)** | **0.28 (0.28)** | **33.22 (< .001)** | 0.03 (0.27) | 0.04 (0.27) | 0.10 (.752) |
| **Energy velocity** | **0.00 (0.00)** | **0.00 (0.00)** | **13.45 (< .001)** | 0.00 (0.00) | 0.00 (0.00) | 0.13 (.716) |
| ***f*_o_ kurtosis** | 5.15 (1.55) | 4.78 (1.72) | 0.99 (.322) | 4.75 (1.19) | 5.27 (1.75) | 2.15 (.147) |
| ***f*_o_ SD** | **18.00 (7.59)** | **25.20 (14.30)** | **7.92 (.006)** | **16.49 (4.43)** | **20.23 (8.57)** | **5.18 (.026)** |
| ***f*_o_ skewness** | 1.05 (0.34) | 1.17 (0.40) | 2.22 (.141) | 1.11 (0.33) | 1.14 (0.50) | 0.11 (.742) |
| ***f*_o_ velocity** | 1.66 (0.73) | 1.87 (0.80) | 1.46 (.230) | **1.41 (0.39)** | **1.73 (0.54)** | **8.42 (.005)** |
| **Intensity kurtosis** | 11.96 (6.49) | 9.90 (6.29) | 2.07 (.154) | 10.09 (6.38) | 9.19 (6.61) | 0.37 (.546) |
| **Intensity SD** | 0.03 (0.01) | 0.03 (0.01) | 0.79 (.376) | 0.04 (0.01) | 0.04 (0.01) | 0.02 (.886) |
| **Intensity skewness** | **2.32 (0.59)** | **2.00 (0.76)** | **4.22 (.043)** | 2.06 (0.76) | 1.94 (0.72) | 0.49 (.486) |
| **LHR kurtosis** | 2.63 (0.38) | 2.70 (0.27) | 0.90 (.345) | **2.62 (0.39)** | **2.83 (0.49)** | **4.27 (.042)** |
| **LHR** | 26.42 (3.25) | 26.97 (4.14) | 0.44 (.510) | 27.46 (3.11) | 27.59 (5.37) | 0.01 (.907) |
| **LHR SD** | **9.17 (0.97)** | **9.85 (1.16)** | **8.20 (.005)** | 9.17 (1.76) | 9.03 (1.58) | 0.13 (.718) |
| **LHR skewness** | -0.46 (0.19) | -0.55 (0.25) | 3.62 (.061) | -0.43 (0.24) | -0.48 (0.35) | 0.45 (.506) |
| **Pause Duration** | 1.40 (0.49) | 1.58 (0.54) | 2.42 (.124) | 1.56 (0.52) | 1.46 (0.85) | 0.38 (.540) |
| **Pause Duration SD** | 1.32 (0.62) | 1.37 (0.80) | 0.10 (.747) | 1.35 (0.50) | 1.36 (1.04) | 0.00 (.961) |
| **Pause rate** | 0.44 (0.12) | 0.47 (0.14) | 0.60 (.440) | 0.48 (0.14) | 0.46 (0.14) | 0.38 (.537) |
| **Speech rate** | 1.69 (0.32) | 1.84 (0.52) | 2.29 (.134) | 1.71 (0.49) | 1.73 (0.42) | 0.04 (.848) |
| **Talking rate** | 0.56 (0.12) | 0.53 (0.14) | 0.60 (.440) | 0.52 (0.14) | 0.54 (0.14) | 0.38 (.537) |
| **MFCC1** | -12.00 (0.78) | -11.76 (0.75) | 1.82 (.181) | -11.94 (0.65) | -11.74 (0.72) | 1.50 (.225) |
| **MFCC2** | 2.42 (0.32) | 2.41 (0.48) | 0.02 (.898) | 2.52 (0.39) | 2.56 (0.54) | 0.11 (.741) |
| **MFCC3** | -0.13 (0.47) | 0.01 (0.37) | 2.15 (.146) | **-0.01 (0.29)** | **-0.24 (0.26)** | **14.48 (< .001)** |
| **MFCC4** | **0.44 (0.28)** | **0.58 (0.20)** | **7.11 (.009)** | 0.66 (0.36) | 0.49 (0.26) | 6.25 (.015) |
| **MFCC5** | 0.18 (0.20) | 0.23 (0.28) | 0.84 (.362) | **0.18 (0.19)** | **0.29 (0.21)** | **5.37 (.023)** |
| **MFCC6** | **-0.02 (0.20)** | **0.06 (0.13)** | **4.34 (.041)** | 0.03 (0.13) | 0.02 (0.19) | 0.13 (.720) |
| **MFCC7** | -0.14 (0.15) | -0.11 (0.16) | 0.63 (.430) | -0.12 (0.14) | -0.17 (0.12) | 3.05 (.085) |
| **MFCC8** | -0.12 (0.19) | -0.11 (0.11) | 0.20 (.656) | **-0.17 (0.15)** | **-0.04 (0.12)** | **19.41 (< .001)** |
| **MFCC9** | **-0.06 (0.13)** | **-0.13 (0.11)** | **6.62 (.012)** | **-0.12 (0.17)** | **0.03 (0.20)** | **12.37 (.001)** |
| **MFCC10** | -0.06 (0.13) | -0.12 (0.13) | 3.16 (.079) | -0.03 (0.10) | -0.02 (0.13) | 0.04 (.849) |
| **MFCC11** | -0.05 (0.12) | -0.08 (0.06) | 2.22 (.140) | -0.05 (0.14) | -0.05 (0.12) | 0.00 (.986) |
| **MFCC12** | 0.02 (0.13) | -0.01 (0.09) | 2.21 (.141) | **-0.07 (0.06)** | **0.02 (0.13)** | **14.28 (< .001)** |
| **MFCC13** | -0.12 (0.09) | -0.08 (0.13) | 2.84 (.096) | -0.09 (0.09) | -0.03 (0.15) | 3.51 (.065) |

Note: Differences that are significant at α = .05 are bolded. See Table S1 for description of speech feature names.

| 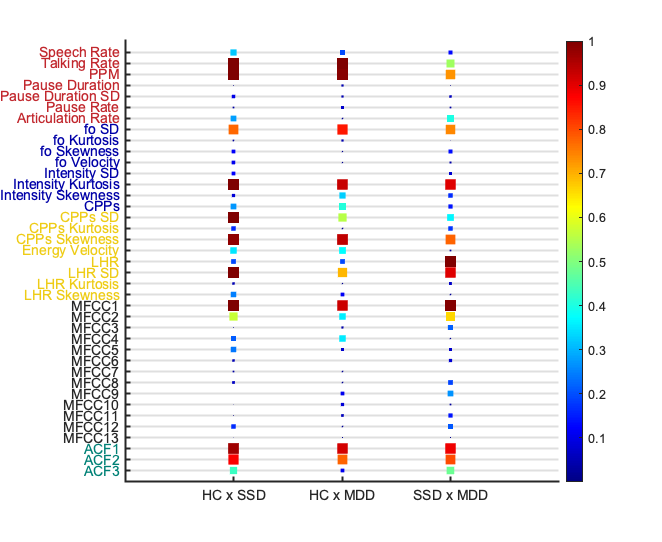 |
| --- |
| Figure S1. Summary of relative feature importance across the three pair-wise machine learning models with the linear support vector machine kernel. Larger boxes and darker red color relate to higher feature importance. For each model there were twenty participants per group (HC, healthy controls; SSD, schizophrenia spectrum disorder; MDD, major depressive disorder) and four speech samples per participant. |
| 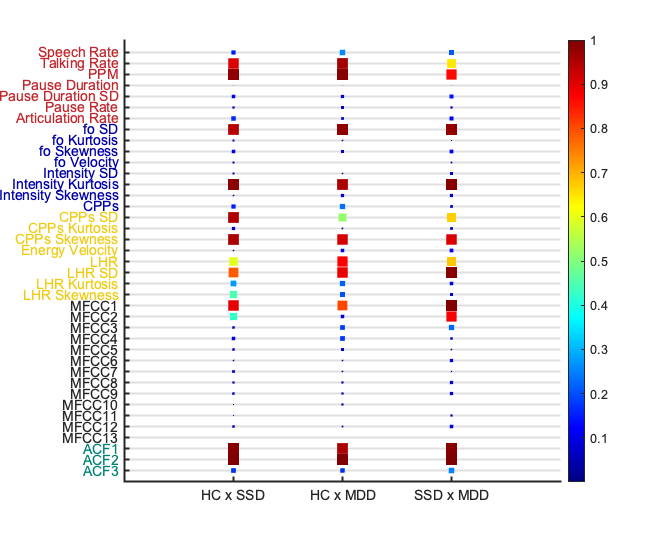 |
| Figure S2. Summary of relative feature importance across the three pairwise machine learning models with the 2-degree polynomial support vector machine kernel. Larger boxes and darker red color relate to higher feature importance. For each model there were twenty participants per group (HC, healthy controls; SSD, schizophrenia spectrum disorder; MDD, major depressive disorder) and four speech samples per participant. |
| 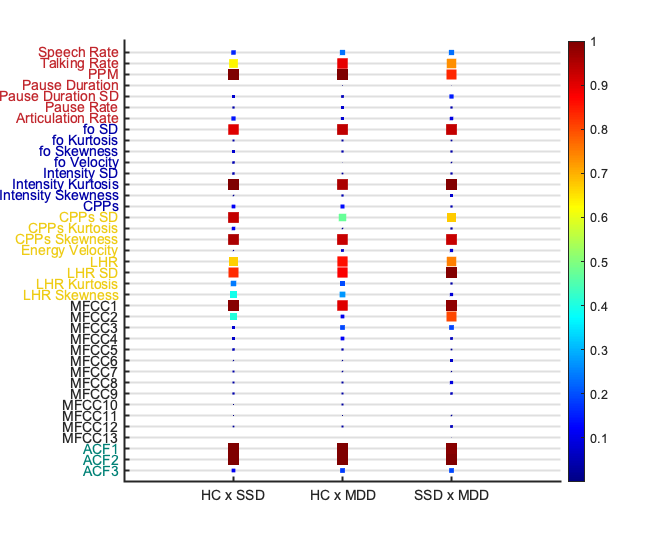 |
| Figure S3. Summary of relative feature importance across the three pairwise machine learning models with the 3-degree polynomial support vector machine kernel. Larger boxes and darker red color relate to higher feature importance. For each model there were twenty participants per group (HC, healthy controls; SSD, schizophrenia spectrum disorder; MDD, major depressive disorder) and four speech samples per participant. |
| 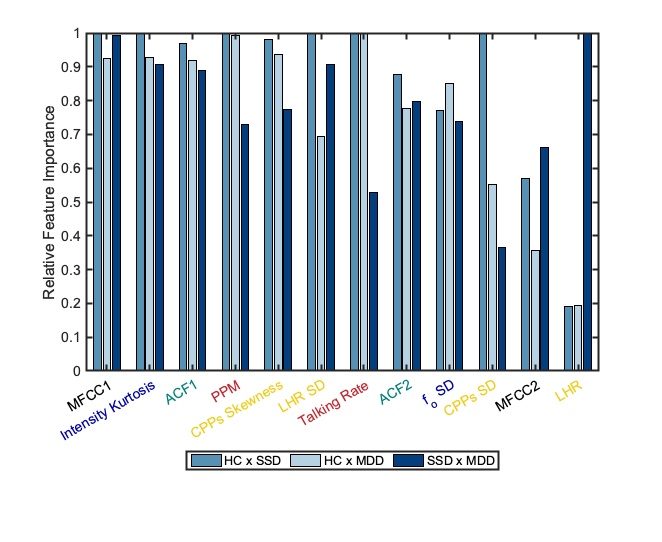 |
| Figure S4. Comparison of the top 25% of features ranked by aggregate feature importance across all pairwise models with the linear support vector machine kernel. For each model there were twenty participants per group (HC, healthy controls; SSD, schizophrenia spectrum disorder; MDD, major depressive disorder) and four speech samples per participant. |

| 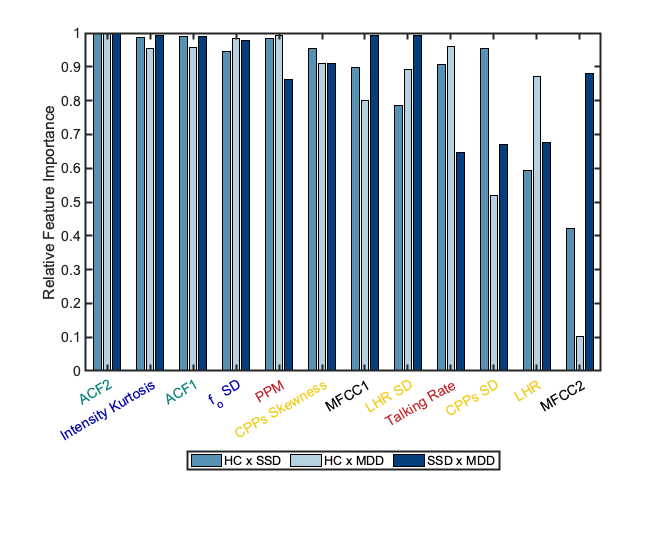 |
| --- |
| Figure S5. Comparison of the top 25% of features ranked by aggregate feature importance across all pairwise models with the 2-degree polynomial support vector machine kernel. For each model there were twenty participants per group (HC, healthy controls; SSD, schizophrenia spectrum disorder; MDD, major depressive disorder) and four speech samples per participant. |

References for Supplementary Material

1. Low DM, Bentley KH, Ghosh SS. Automated assessment of psychiatric disorders using speech: A systematic review. Laryngoscope Investig Otolaryngol. 2020;5(1):96-116. doi:10.1002/lio2.354.

2. Matsumoto K, Kircher TTJ, Stokes PRA, Brammer MJ, Liddle PF, McGuire PK. Frequency and neural correlates of pauses in patients with formal thought disorder. Front Psychiatry. 2013;4:127. doi:10.3389/fpsyt.2013.00127.

3. Quatieri TF, Malyska N. Vocal-source biomarkers for depression: A link to psychomotor activity. In: Thirteenth Annual Conference of the International Speech Communication Association; 2012.

4. Heman-Ackah YD, Heuer RJ, Michael DD, et al. Cepstral peak prominence: A more reliable measure of dysphonia. Ann Otol Rhinol Laryngol. 2003;112(4):324-333. doi:10.1177/000348940311200406.

5. Silva WJ, Lopes L, Galdino MKC, Almeida AA. Voice acoustic parameters as predictors of depression. J Voice. 2021. doi:10.1016/j.jvoice.2021.06.018.

6. Awan SN, Roy N. Toward the development of an objective index of dysphonia severity: A four-factor acoustic model. Clin Linguist Phon. 2006;20(1):35-49. doi:10.1080/02699200400008353.

7. Espy-Wilson C, Lammert AC, Seneviratne N, Quatieri TF. Assessing neuromotor coordination in depression using inverted vocal tract variables. In: Interspeech 2019. ISCA: ISCA; 2019:1448-1452.

8. Giannakopoulos T. A method for silence removal and segmentation of speech signals, implemented in Matlab. University of Athens, Athens. 2009; 2.

9. Selting M, Auer P, Barden B, et al. Gesprächsanalytisches Transkriptionssystem (GAT). Linguistische Berichte. 1998:91-122.

10. Praat: doing phonetics by computer: [Computer program]; 2022.

11. Kapsner-Smith MR, Díaz-Cádiz ME, Vojtech JM, et al. Clinical cutoff scores for acoustic indices of vocal hyperfunction that combine relative fundamental frequency and cepstral peak prominence. J Speech Lang Hear Res. 2022;65(4):1349-1369. doi:10.1044/2021_JSLHR-21-00466.

12. Maryn Y, Weenink D. Objective dysphonia measures in the program Praat: smoothed cepstral peak prominence and acoustic voice quality index. J Voice. 2015;29(1):35-43. doi:10.1016/j.jvoice.2014.06.015.

13. Auditory Toolbox: A Matlab toolbox for auditory modeling work; 1998.

14. Ittichaichareon C, Suksri S, Yingthawornsuk T, eds. Speech recognition using MFCC; 2012; 9.

15. Siriwardena YM, Espy-Wilson C, Kitchen C, Kelly DL. Multimodal approach for assessing neuromotor coordination in schizophrenia using convolutional neural networks. In: Hammal Z, Busso C, Pelachaud C, Oviatt S, Salah AA, Zhao G, eds. Proceedings of the 2021 International Conference on Multimodal Interaction. New York, NY, USA: ACM; 2021:768-772.

16. IBM Corp. (2017). IBM SPSS Statistics for Windows. Armonk, NY: IBM Corp.
