# Supplementary figures and images for: Relative importance of speech and voice features in the classification of schizophrenia and depression
Source: Transl Psychiatry. 2023 Sep 19;13:298. doi: 10.1038/s41398-023-02594-0 (PMC10509176; doi:10.1038/s41398-023-02594-0)

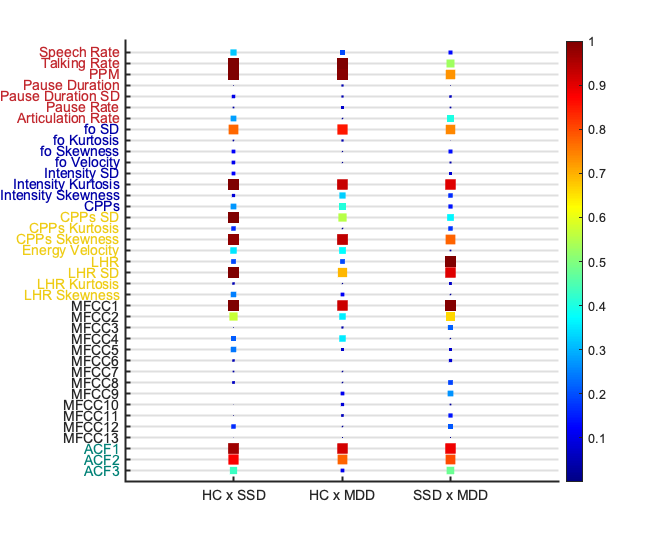

Supplement: Supplementary file 2 — Figure_S1 [file 41398_2023_2594_MOESM2_ESM.png]

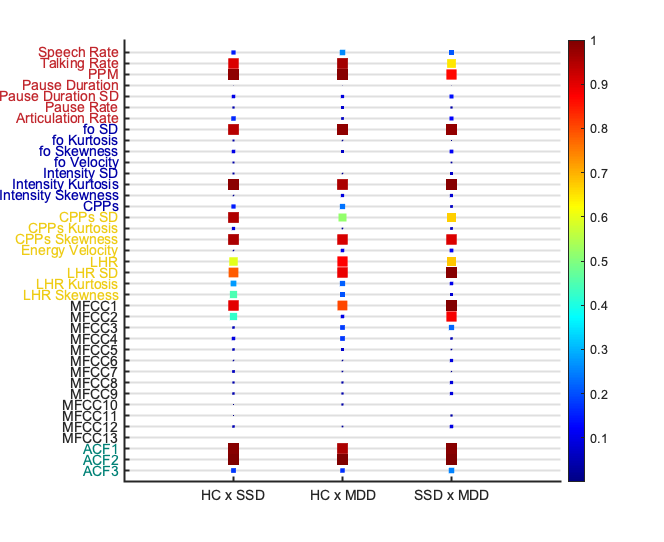

Supplement: Supplementary file 3 — Figure_S2 [file 41398_2023_2594_MOESM3_ESM.png]

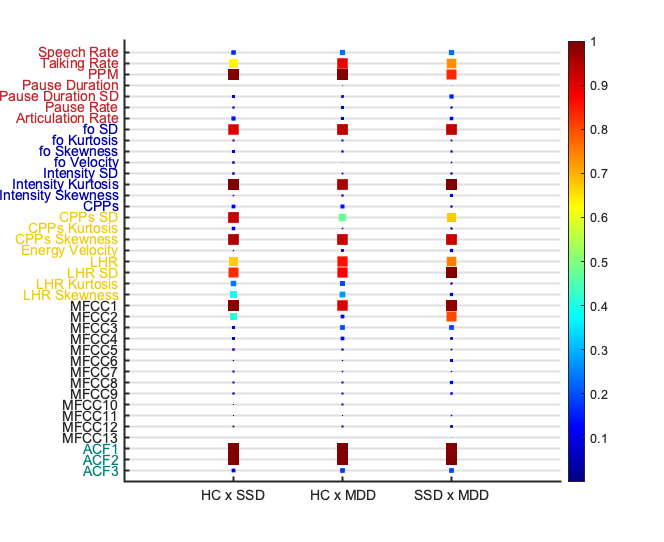

Supplement: Supplementary file 4 — Figure_S3 [file 41398_2023_2594_MOESM4_ESM.png]

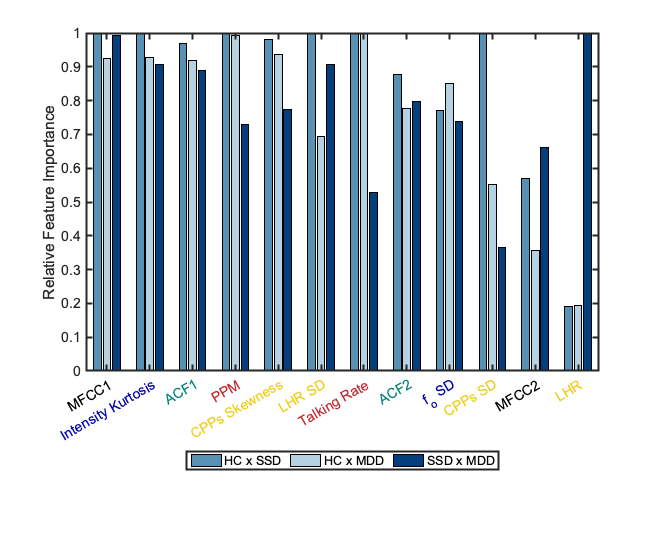

Supplement: Supplementary file 5 — Figure_S4 [file 41398_2023_2594_MOESM5_ESM.png]

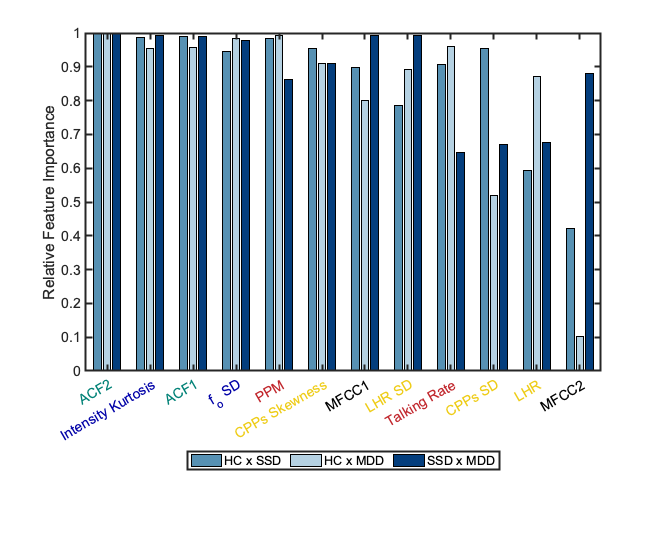

Supplement: Supplementary file 6 — Figure_S5 [file 41398_2023_2594_MOESM6_ESM.png]
